# Systematic Surface Phase Transition of Ag Thin Films by Iodine Functionalization at Room Temperature: Evolution of Optoelectronic and Texture Properties

**DOI:** 10.1038/srep21439

**Published:** 2016-02-22

**Authors:** Muhammad Y. Bashouti, Razieh Talebi, Thaer Kassar, Arashmid Nahal, Jürgen Ristein, Tobias Unruh, Silke H. Christiansen

**Affiliations:** 1 Max-Planck Institute for the Science of Light, Günther-Scharowsky-Str. 1, D-91058, Erlangen, Germany; 2 Department of Physics, Faculty of Science, University of Isfahan, Hezar Jerib, 81746-73441, Isfahan, Iran; 3 Crystallography and Structure Physics, Physics Department, Friedrich-Alexander-Universität Erlangen-Nürnberg Staudtstraße 3, 91058 Erlangen, Germany; 4 Department of Physics, Photonic Materials Research Laboratory, University of Tehran, 14399-55961, Tehran, Iran; 5 Department of Physics, Chair of Laser Physics, Universität Erlangen-Nürnberg, Staudtstr. 1, D-91058 Erlangen, Germany; 6 Institute of Nanoarchitectures for energy conversion, Helmholtz-Center Berlin (HZB), Hahn-Meitner-Platz 1, D-14109 Berlin, Germany; 7 Physics Department, Freie Universität Berlin, Arnimallee 14, 14195 Berlin, Germany

## Abstract

We show a simple room temperature surface functionalization approach using iodine vapour to control a surface phase transition from cubic silver (Ag) of thin films into wurtzite silver-iodid (β-AgI) films. A combination of surface characterization techniques (optical, electronical and structural characterization) reveal distinct physical properties of the new surface phase. We discuss the AgI thin film formation dynamics and related transformation of physical properties by determining the work-function, dielectric constant and pyroelectric behavior together with morphological and structural thin film properties such as layer thickness, grain structure and texture formation. Notable results are: (i) a remarkable increase of the work-function (by 0.9 eV) of the Ag thin layer after short a iodine exposure time (≤60 s), with simultaneous increase of the thin film transparency (by two orders of magnitude), (ii) pinning of the Fermi level at the valance band maximum upon iodine functionalization, (iii) 84% of all crystallites grain were aligned as a result of the evolution of an internal electric field. Realizing a nano-scale layer stack composed of a dielectric AgI layer on top of a metallic thin Ag layer with such a simple method has some technological implications e.g. to realize optical elements such as planar optical waveguides.

Silver thin films (AgTF) have optoelectronic properties that can be exploited essentially when functionalization and nanoscale patterning permit a precise tuning of their physical properties^1^,^2^. These Ag TFs usually show a high reflectivity, a good conductivity, low polarization from the far infrared (IR) to the visible region (limited to ≥400 nm)^3^,^4^. Applications so far reside in the areas of transparent conductive coatings, transparent heat mirrors and other optical applications^5^,^6^,^7^,^8^,^9^. To use AgTFs for optical wave-guiding, the decay length of evanescent electromagnetic waves needs to be substantially reduced as can be realized by the deposition of a dielectric layers on the AgTF surface^10^,^11^,^12^,^13^,^14^,^15^,^16^. A promising approach to control the formation of such dielectric thin layers on top of AgTFs is to use inorganic reactants such as halides, (X = F, Cl, Br, I) which permit a systematic tuning of the surface properties of AgTFs^17^. Realizing a controlled dielectric coating on AgTFs with nanoscale thicknesses permits the control of the propagation of electromagnetic waves within the AgTFs. After a proper adaption of a dielectric layer on AgTFs, a reduction in transmission losses of far IR light could already be revealed^16^. In the present work, we report on a comparably simple approach to convert the top layer of the metal AgTF into a dielectric AgI TF and thereby to transform the surface phase from cubic to wurtzite phase within a short time of iodine vapour exposure (0–180 s) at room temperature. In this approach, the iodine reactants are obtained via the equilibrium dissociation of iodine vapour (2Ag + I_2_ → 2AgI) and transferred vertically through a membrane to the AgTF surface. The surface phase transition properties (electronic, optical and structural properties) are monitored as a function of the iodine exposure time using a combination of total photoelectron yield spectroscopy (TPYS), x-ray photoemission spectroscopy (XPS) *in Suite* with Kelvin-probe (KP), integration sphere and Grazing Incidence X-ray Diffraction (GIXD). We show that a systematic control of the surface phase transition can be achieved by simply adjusting the iodine exposure time. The surface phase transition from cubic silver (Ag) to wurtzite silver-iodide (β-AgI) was confirmed by GIXD. The results show the increase of work-function by 0.9 eV and transparency by two orders of magnitude and pinning the Fermi level down to the valance band maximum. The TPY spectra show the development of a semiconducting pyroelectric layer (β-AgI) that gives rise to a gradual shift of the photoelectron excitation threshold and the Fowler function. 84% of all crystallites forming the β-AgI TF turn out to be aligned along the [001] zone axis after less than 60 s of iodine exposure. This texture formation may most probably be due to the built up of an internal electrical field as the β-AgI TF grows and the transition from cubic Ag to wurtzite β-AgI takes place. The textured grain alignment in the β-AgI TF is further supported by GIXD that show a preferred surface normal orientation along a [001] zone axis.

## Results and Discussion

**Figure 1:**
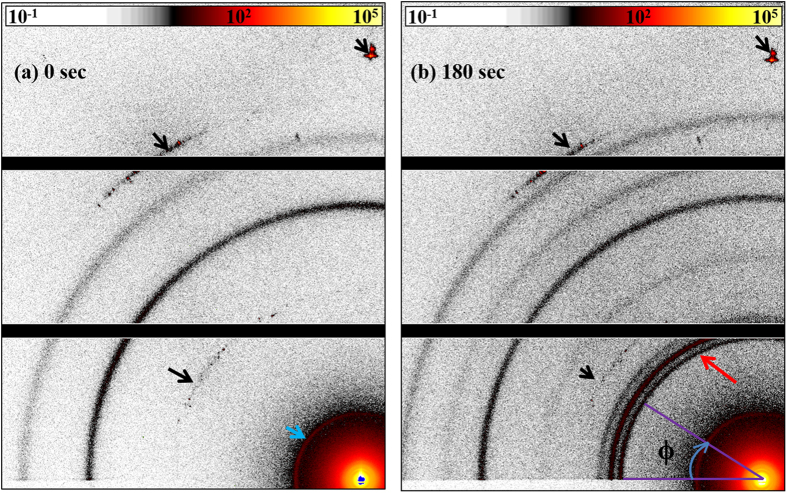
GIXD detector images. GIXD detector images collected for (**a**) the native AgTF and (**b**) for the same film after an exposure to iodine for 180 s. Some scattering artefacts originating from the Si substrate at sample edges (black arrows) or from the detector itself (blue arrow) are indicated. The red arrow points to the intersection line of the Debye-Scherrer cone of the (100) Bragg reflection of β-AgI with the detector plane.

We have followed systematically the surface phase transfer in a series of grazing incidence x-ray diffraction (GIXD) experiments. A textured growth of β-AgI (wurtzite crystal structure) with a preferential orientation of the 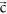 axis perpendicular to the surface of the substrate could be observed by these measurements. Figure 1 displays two GIXD patterns which demonstrate the evolution of AgI upon exposure to iodine vapor: Fig. 1a for the native AgTF and Fig. 1b for the same TF after an exposure to iodine for 180 s. The full set of patterns for all exposure times (0, 3 s, 6 s, 20 s, 40 s, 60 s, 120 s and 180 s) is visualized in the supplementary information, Figure S1.

**Figure 2:**
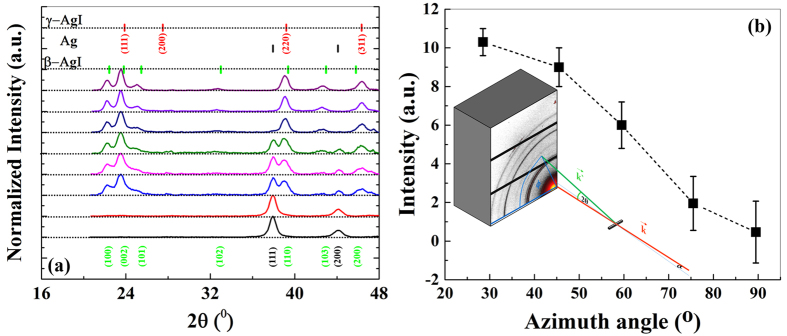
The azimuthally averaged detector GIXD patterns of the native Ag TF. (**a**) Azimuthally averaged GIXD patterns of the native Ag thin film and after progressive exposure to iodine. The curves are labelled by the exposure times. The tick marks indicate the Bragg angles of Ag (black), β-AgI (green) and γ-AgI (red) as indicated in SI. (**b**) Azimuthal distribution of the intensity scattered from (100) planes of the β-AgI crystallites after an exposure to iodine for 1800 s. The azimuth angle ϕ in this case is equal to the angle between <100> direction and the substrate. The inset visualizes the scattering geometry of the GIXD experiment with the incident x-ray beam (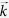) and the scattered beam (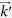). In our experiment 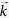 is inclined with respect to the sample surface by 0.2°.

The azimuthally averaged detector GIXD patterns of the native Ag TF and after progressive exposure to iodine is shown in Fig. 2. Figure 2a shows the azimuthally averaged detector patterns with a comparison of the diffraction data with the Bragg peak positions computed for fcc Ag, α-AgI, β-AgI and γ-AgI, respectively. The absence of the distinctive α-AgI (200) Bragg reflection at 2θ = 35° reveal that (not surprisingly) the high temperature super-ionic conductive α-AgI phase has not been formed. The diffraction patterns can be nicely assigned to the wurtzite β-AgI phase and marginal remaining of Ag. If any γ-AgI has been formed it is only a minor amount which gives rise to the solely low intensity of the γ-AgI (200) at 27.5°. Thus it can be concluded that β-AgI is almost exclusively formed by the iodine exposure. The evolution of area of the peaks belonging to the β-AgI phase clearly shows that surface phase is rapidly transferred from Ag TF into β-AgI after exposure to iodine. Already an exposure time of three seconds to iodine vapour is enough for partial surface phase transfer. The gradual transition of the Ag TF into β-AgI top layer is mainly completed after 180 seconds of exposure to iodine vapour. Figure 2b shows that the azimuthal distribution of the intensity of β-AgI reflections is not uniform indicating a preferred orientation of the silver iodine crystallites. The data traces in this figure were obtained by extracting the detector intensities along the arc covering the β-AgI (100) reflection. The data points result from a dedicated fitting routine yielding in the deconvolution of the Bragg peaks in the angular range between 20° and 90° in an azimuthal sector of 3° around some selected Φ angles. Obviously, the (100) Bragg peak intensity strongly varies with Φ with a pronounced intensity maximum for small Φ. Already from this observation a preferential orientation of the β-AgI crystallites in the films can be concluded. The normal vectors of the (100) lattice planes of the crystallites are preferentially oriented in parallel to the film surface. The relative drop of the curve in Fig. 2b can be taken as a qualitative measure for the texture of the β-AgI crystallites with the hexagonal 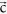 axis perpendicular to the sample surface. This texture is clearly significant for the 180 s films and even more pronounced after 1800 s of iodine exposure (Figure S2).

**Figure 3:**
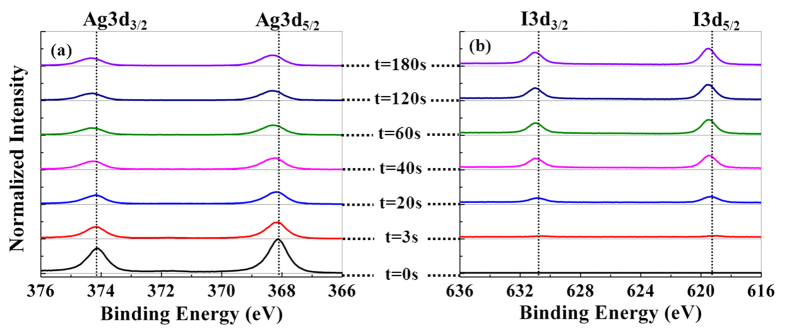
XP spectra of Ag3d and I3d. High resolution scans of XP spectra for the different accumulated iodine exposure times for: (**a**) Ag_3d_ (366–376 eV), and (**b**) I_3d_ (616–636 eV). The complete deconvolution data of Ag3d and I3d can be found in Table S1. One example of the deconvolution is explained in SI and illustrated in Figure 4S.

A full scan survey x-ray photoelectron spectrum (XPS) of the AgTFs before (t = 0 s) and after the full exposure time (t = 180 s) to iodine vapor can be found in Figure S3. The lack of C1s, O1s and S2p signals in the XPS data, which would have appeared at 285 ± 1 eV, 532 ± 1 eV and 165 ± 2 eV, respectively, confirms that the iodized surfaces were not contaminated or oxidized or sulfurized after the iodine exposure. Interestingly enough, even small traces of hydrocarbon contaminants on the initial AgTFs witnessed by C1s and O1s lines were almost no longer present after iodine exposure. Figure 3 presents the spin-orbit doublets of the 3d core level spectra of silver (616–636 eV) and of iodine (366–376 eV) after each step of iodine exposure. One example for the Ag3d and I3d deconvolution is illustrated in Figure S4. The spectra are representative for the stoichiometry of the AgTFs within a surface-near layer of the extension of the probe depth of XPS. This probe depth λ = λ_о_∙sinγ is given by the inelastic scattering length λ_о_ of the photo excited electrons in matter (~2.5 nm) and the pick-up angle γ of the photoelectrons with respect to the surface plane (35° in our experiment) resulting in λ = 1.4 nm^18^.

**Figure 4:**
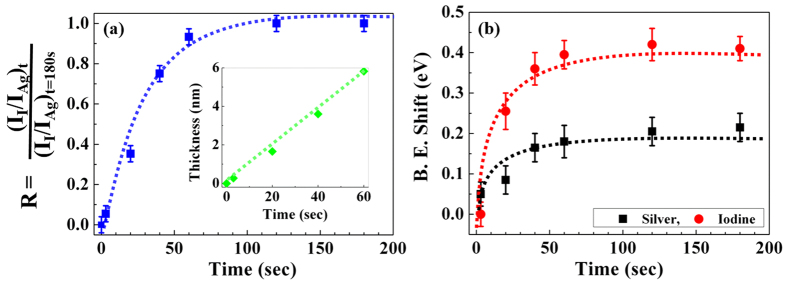
(**a**) Two model layer and shift in 3d emission as function of time. Ratio of iodine to silver photoemission intensities normalized to the respective saturation value at (t = 180 s); inset is the thickness of the β-AgI top layer as evaluated from the XP intensity ratios (see SI). (**b**) The binding energy shift of Ag_3d_ and I_3d_ core lines as function of iodine exposure.

The spectra show a continuous increase of the iodine and an accompanying decrease of the silver signal with longer iodine exposure: both reaching characteristic saturation values after about 60 s. The straight forward interpretation of a sequence of spectra between 0 s and 180 s of iodine exposure shows a gradual transformation of the AgTF into β-AgI thin film as supported from the aforementioned GIXD results (section 1) and optoelectronic properties in the next sections^19^,^20^. Accompanying the change in surface stoichiometry, we observe a systematic shift of both the Ag and the I core level doublets towards higher binding energies (*cf.* Fig. 3). For a quantitative evaluation, we have fitted the spectra and plotted the intensity ratio (R) of the iodine and the Ag core lines normalized to the respective saturation values at t = 180 s as shown in Fig. 4a and the line shift of the spectra as shown in Fig. 4b. Additionally, we have added the evolution of the β-AgI top-layer thickness for the first four time slots of iodine exposure (inset Fig. 4a) as it results from an analysis of the stoichiometry in the framework of an effective two layer model. Initially (t = 0 s, no iodine exposure), the sample consists of a silver layer of thickness d_2_ (t = 0 s) = d_0_ on top of the silicon substrate only. In the course of iodine exposure, Iodine diffuses into the sample and transforms the Ag TF gradually to β-AgI, starting from the surface and proceeding towards the bulk. Hence, the most general case for the stack during this transformation is a residual layer of metallic silver of thickness d_2_ < d_0_ buried under a layer of AgI of thickness d_1_. From both layers, we generally expect Ag photoelectrons, whereas Iodine photoelectrons are exclusively assigned to the top-layer (Fig. 4a). Assuming the same inelastic scattering depth λ for both materials under consideration, we may write the photoelectron intensities as:


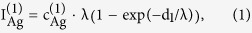


and


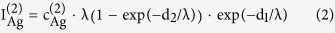


The constants *c* contains the atomic photoionization cross sections, all relevant spectrometer constants and the silver atom density of the respective layer. Only this latter quantity differs between the two silver containing layers and thus we may re-write the second equation as:


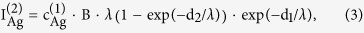


where the constant B = 4.03 is the ratio of the silver atomic density in metallic Ag and AgI^21^,^22^. With this transformation we may write the total silver photoemission intensity from both layers as:


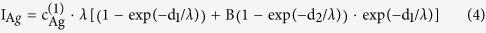


The iodine photoemission intensity can be expressed as:


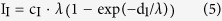


Thus, the ratio (R) between the intensities of the silver and iodine is:


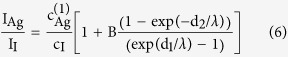


Since 
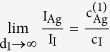
 we may normalize the intensity ratio to its asymptotic limit and obtain finally the ratio between silver and iodine which express the thickness of the nano-phase:


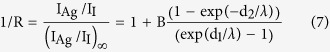


1/R is the reciprocal of the data plotted in Fig. 4a. As long as the remaining silver layer is sufficiently thick, i.e. 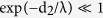, it can be easily transformed to yield the AgI layer thickness d_1_:


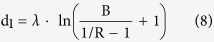


In the transition range of Fig. 4a, i.e. for accumulated exposure times between 3 and 60 s, the XPS data following this analysis yield silver reliable iodide layer thicknesses of that are listed the full text (inset in Fig. 4a). Note that even for the last data point involved, the remaining silver layer thickness d_2_ allows the approximation made above: due to the fourfold lower silver atom density compared to the metallic phase, the growth of 5.8 nm β-AgI only consumes about 1.5 nm of the Ag layer, and the residual d_2_ = 8.5 nm certainly fulfil 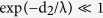. For the XPS intensity ratios approaching saturation in Fig. 4a (t = 180 s for instance), the two-model-layer analysis gives no result for the β-AgI layer thickness within reasonable error limits (approximate thickness can be followed by AFM as shown in Figure S5 and Table S2). The shift of the iodine core lines accompanying the iodine exposure (full circles in Fig. 4b) is assigned to a gradual shift of the Fermi level relative to the valance band edges of the β-AgI semiconductor as will be discussed later (section 4). The shift of the Ag3d core lines is in fact more complicated since here the photoelectrons from the β-AgI over-layer undergo a chemical shift due to the formation of a ionic compound (β-AgI) (full square in Fig. 4b). From the data we infer a growth rate for the β-AgI top-layer of about 0.1 nm/s.

**Figure 5:**
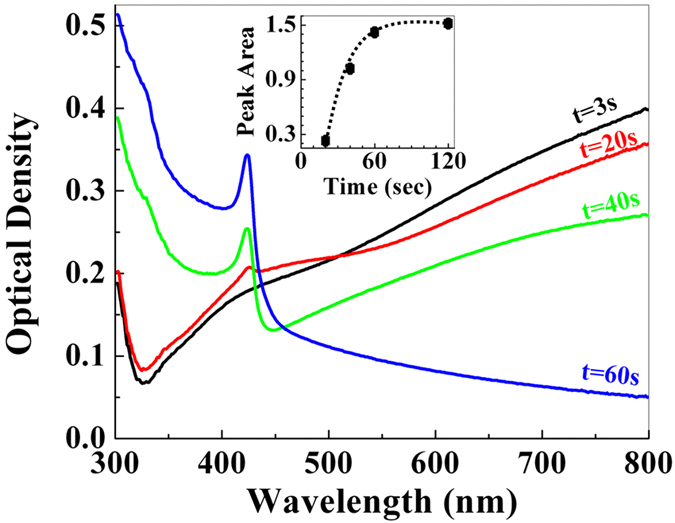
Optical density spectrum of the AgTF. Optical density spectrum of the AgTF as a function of the iodine exposure time. The inset shows the area under the absorption peak at 420 nm characteristic for AgI.

As pointed out above, only the first few nanometres of the AgTF are accessible to photoelectron spectroscopy. In order to characterize the evolution of the nano-phase films for exposure times longer than about 60 s we have measured the optical density spectra in the visible and near UV range of identically processed AgTFs on glass substrates as a function of iodine exposure times (Fig. 5). The initial film and the one exposed to iodine for 3 s are identical and increase over the whole visible range characteristic for a Ag TF (see Figure S6). During further iodine exposure (20 s, 40 s, 60 s) the spectra systematically change and finally the optical density is decreasing with wavelength and exhibit marked resonant absorption at 420 nm that is characteristic for AgI^23^,^24^,^25^. The area under the absorption peak at 420 nm is indicative of the formation of nano-phase i.e. β-AgI top-layer and increases systematically with the iodine exposure. With the bare eye a change in the film transparency from opaque to transparent is discernible upon iodine exposure (see Figure S7). Also the evolution of the optical density spectra indicates the transformation of the surface layer of AgTF into β-AgI until the entire AgTFs are almost completely transformed which supported by the GIXD measurements^26^,^27^.

**Figure 6:**
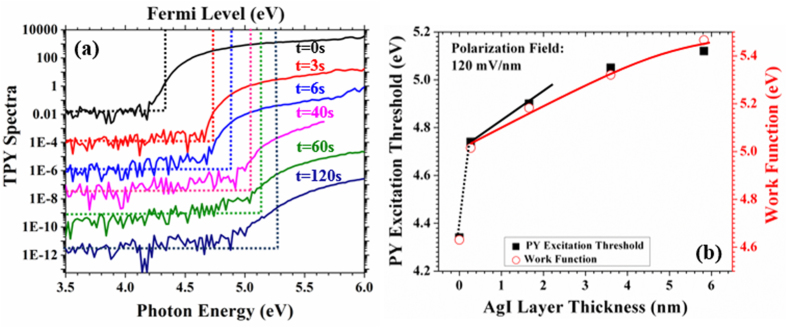
TPY spectra of the Ag TF as a function of the iodine exposure. (**a**) The vertical dashed lines represent the Fermi-level of the AgTF with respect to iodine exposure. The horizontal dash lines represent the base line of each individual TPY spectrum. The TPYS after 3 s and 180 s were not included since they looks identical to 6 s and 180 s respectively. (**b**) photon-yield threshold energies as obtained from Fowler function fitting (squares, left hand scale) and work functions (circles, right hand scale) as measured by the Kelvin method (KP) as a function of AgI top layer thickness.

Electronically, the surface-phase transformation of the AgTF to β-AgI is supported by a characteristic change in photoelectron behaviour and a change in work function. For the former, high dynamic total photoelectron yield spectra (TPYS) were taken^28^,^29^,^30^. For the latter the contact potential difference (CPD) with respect to a calibrated Au probe was monitored. Both experiments were performed *in situ* accompanying the XPS measurements, i.e. on the very same samples. Figure 6a shows the TPY spectra versus photon energy (*hv*) of the bare AgTF and of the same sample after five selected time frames of iodine exposure. The TPY spectra strongly shifted to higher photon energies with increasing iodine exposure and the photoemission quantum efficiency decreases several orders of magnitude in the course of iodine exposure. Moreover, the shape of the spectra clearly changes. For exposure times up to 6 s the TPY spectra show the Fowler characteristics of metals proving that the photoelectrons are excited from the AgTF in these three cases (0 s, 3 s and 6 s)^29^. From Fowler fitting we extracted the excitation threshold energy of the TPY spectra and plot them for iodine exposure times up to 60 s together with the work function obtained from the CPD as shown in Fig. 4b. (The complete work function data set is shown in Table S3). Both data sets are identical within the experimental error up to an offset of 0.19 eV. This offset is a consequence of the tailing of the Fowler function imaging the Fermi-Dirac occupation statistics of the electrons in metals^29^.

**Figure 7:**
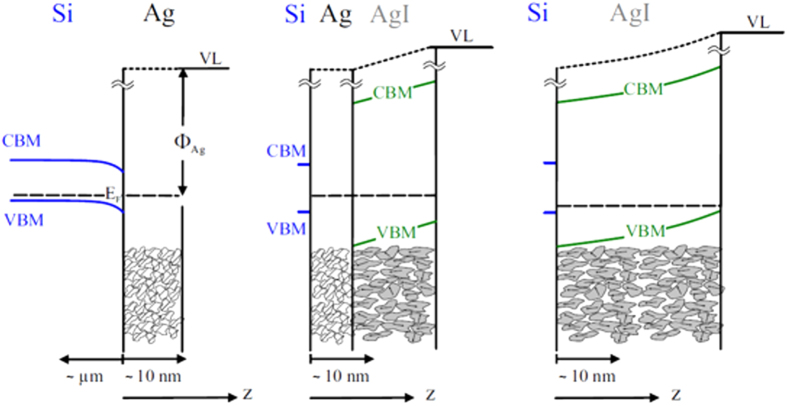
Band Diagrams of Ag TF. Surface band diagrams for the evaluation of a β-AgI top layer onto a AgTF on a p-type silicon substrate. Left: Si-Ag Schottky junction; middle: partially β-AgI-converted AgTF; right: Si- β-AgI interface after complete reaction of Ag with I so that no pure Ag interlayer is left.

The initial AgTFs show a work function of 4.63 eV that as in excellent agreement with average reported literature values of 4.65 ± 0.15 eV for principle crystallographic surfaces of clean single-crystalline silver^31^,^32^,^33^. After 3 s of iodine exposure, the work-function of the AgTF has increased by 0.4 eV. The effective β-AgI top-layer thickness obtained from the XPS analysis for that film was 2.7 Å which is less than the sum of the atomic radii of Ag^**+**^ (1.00 Å) and I^**−**^ (2.20 Å). We are thus beyond monolayer transition and no crystalline compound has been formed so far. Nevertheless, we observe a strong increase of the work function and the photon-yield threshold by 0.4 eV. One mechanism can potentially be responsible for this effect: an initial ionic double layer of Ag and I is deposited on the Ag surface and the increase of work function and photoelectron excitation threshold is due to the potential step associated with the dipole moment of that layer. With further iodine exposure (20 s, 40 s, 60 s) the silver grains are more and more converted to β-AgI. The evolution of the wurtzite β-AgI phase with its pronounced texture is in perfect accordance to the pyroelectric polarization of the films as it will be discussed below. The increase of work function and photoelectron threshold energy of the TFs with increasing iodine exposure can be understood by the schematic band diagram presented in Fig. 7. The left hand panel represents the AgTF before iodine exposure, i.e. with the really clean surface. The band diagram shows the Schottky contact as it forms between silver and p-type silicon with a barrier height expected around 0.4 eV^32^. Note the different scales of the depth coordinate on the silver and the silicon side of that sketch.

The middle panel shows the triple layer structure Si/Ag/β-AgI during the transformation of the surface of the AgTF to β-AgI. The band gap of β-AgI is 2.8 eV while the band offset between Ag and β-AgI is unknown^33^. In general, all compounds of the wurtzite crystal structure like β-AgI are pyroelectric, i.e. they exhibit a spontaneous polarization (bulk dipole density) 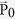 upon heating. In all cases known to the authors so far, 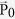 is pointing opposite to the 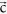 axis of the crystal structure. Following the common sign transition, we will denote by P the component of 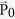 along the 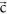 axis, i.e. in the usual case P < 0. We have schematically indicated the polarization of the β-AgI grains by arrows in the schematics of Fig. 5 where we count the depth coordinate z positive from bulk to the surface. Due to the textured growth, an average dipole density 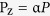 (and P_x_, P_y_ = 0 for symmetry reasons) has to be assigned to the β-AgI TF where 0 < α < 1 is the degree of crystal orientation. Following simple electrostatic concepts, an internal electric field 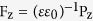 is connected with the spontaneous polarization where ε is the static dielectric constant of the β-AgI layer and ε_0_ the vacuum permittivity. In general the spontaneous polarization of pyroelectric crystals is very difficult to address experimentally, because associated electric fields are neutralized by internal (electrons, holes, charged defects) or external (surface ions) charges that can hardly be controlled quantitatively. Only the *modulation* of the compensating charges upon external perturbation such as strain, temperature variation or the presence of optical phonons can be measured. For quantitative information one has to rely on theory therefore. Quantum mechanical solid state theory of electronic properties is based on periodic boundary conditions which are incompatible with the concept of a bulk dipole density^34^. The spontaneous polarization of wurtzite pyroelectrics is therefore usually inferred by calculating the electrical field discontinuity at the interfaces in a periodic stack of hexagonal wurtzite (h) and cubic zincblende (c) type layers of the respective compound by density functional theory. Since 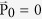 in the cubic zincblende layers for symmetry reasons, 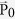 for the wurtzite layers can be directly extracted from that field discontinuity 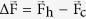 using 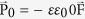. We adopted the most recent DFT calculation of Morgan and Madden for AgI and extracted 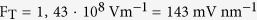 and 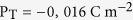 using 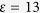 for the compounds of field and polarization parallel to the crystal 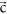 axis^35^. We use the subscript (T) to explicitly mark this as the theoretical value for single crystal β-AgI. This value is about a factor of two to three lower than typical polarizations for II-VI and III-V wurtzite semiconductors^36^. The Photoelectrons associated with the TPY spectra are excited in the AgTF and pass the AgI on their way to the surface. Thus, photoelectrons sense the electronic field due to the polarization. Consequently, the polarization field inside the β-AgI TF raises or lowers the vacuum level for photoelectrons and a corresponding blue shift of the TPY threshold is observed. For β-AgI TFs the effect of free electrons and holes in the conduction band (CBM) and valance band (VBM), respectively, can be ignored and we expect a linear shift of the photon-yield threshold (and of the work function) with β-AgI TF thickness. The blue shift of the yield spectra with increasing the β-AgI TF thickness proves that this electric field points from the substrate side to the surface of our Si/Ag/ β-AgI layer stack. From the slope of the data plot in Fig. 6b for small β-AgI TF thicknesses we can extract the value of F_z_ = 120 mV/nm for this field. The comparison with the calculation yields F_z_ = 0.84F_T_. We can translate this to a textured growth of the β-AgI crystals with 84% preferential orientation of the c-axis from bulk to surface, i.e. along our z-axis in Fig. 7. This is qualitatively in accordance with the textured film morphology as analysed by x-ray diffraction as discussed above. A more detailed and quantitative texture analysing is subject of ongoing research. For thicker layers, the data in Fig. 6a,b show a sub-linear increase with gradual saturation. This can be understood by considering the band diagram in the right hand panel of Fig. 7 where we have sketched a fully converted AgTF on silicon. The upward band bending in the β-AgI layer causes positive space charge due to holes since the Fermi level approaches more and more the valence band maximum^37^. Associated with positive space charge is positive curvature of the bands. Since the field directly at the surface is always given by F_z_ independent of β-AgI TF thickness, this curvature lowers the slope of the potential gradually when going deeper into the film. This explains the sub-linear characteristics in Fig. 6b of the most right band diagram for thicker films. Note that this is the general mechanism by which polarization fields in macroscopic pyroelectrics are screened by internal space charge profiles.

## Conclusion and Outlook

In summary, we have realized AgTFs by sputtering 10 ± 2 nm of Ag on p-type Si. The AgTFs were exposed to iodine vapor at room temperature for systematically varied times between 0 s and 180 s. We observe a gradual transformation of the AgTF to a highly textured β-AgI with 84% preferential 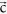 axis orientation relative to the surface normal. Grazing incidence x-ray diffraction, photoelectron emission and threshold energies, and work functions are witnessing this transformation. Thus, the transparency of the AgTFs changes drastically due to the spontaneous polarization of the pyroelectric β-Ag TF. Specifically, when a slab of pyroelectric material is embedded between non-pyroelectric layers the two surfaces inevitably carry a polarization charge with identical magnitude and opposite sign on the two interfaces, and therefore, change the electronic and optical properties of the material/interface which are useful for waveguide applications. Moreover, the AgI layer on top of the bare Ag layer protects the latter from unwanted oxidation.

## Methods

### Sample Preparation

AgTFs were realized by sputtering Ag with a rate of 5.7 Å/s on p-type silicon substrates for electrical and optoelectronic measurements. Identical films were sputtered on glass substrates for comparative optical experiments. In both cases, the initial film thickness of AgTFs was 10 ± 2 nm. Subsequently, the Ag TFs were exposed to iodine vapour for 0–180 s at STP conductions.

### TPYS

TPY spectra were measured between 3.5–6.2 eV with dynamical range of eight orders of magnitude. A 1000-W Xe arc lamp was used as a light source, and the extreme sensitivity was achieved by using an optical double monochromator with a stray light reduction better than 10^−9^. The threshold was determined by fitting to a Fowler-type shape in log scale or by linear fitting in the linear scale^29^.

### KP

The work functions of the samples were determined by measuring the contact potential difference (CPD) with respect to a 3 mm circular and semi-transparent probe by the Kelvin probe method. The work function of this probe was measured and regularly confirmed in calibration experiments with an evaporated Au film whose work function was determined by its photoelectron yield spectra fitted to a Fowler type shape that is valid for metals in a very good approximation. The overall accuracy of this technique can be estimated to about ±30 meV for the work function data obtained.

### XPS

Scan times of up to ~2 h were employed for each scan window and data analysis was performed using the Sigma Probe Advantage software. More information can be found in ref. 18. Precise binding energy positions and intensities were calculated by peak fitting using software package (XPSPEAK version 4.1). Peak fitting solutions were sought for x^2^ < 1, where x^2^ stands for the standard deviation.

### Optical density

The optical density was measured by Cary 5000 Varian UV-Vis-NIR Spectrophotometer with un-polarized light beam.

### Grazing Incidence X-ray Diffraction (GIXD)

GIXD patterns were collected with the highly customized Versatile Advanced X-ray Scattering instrumenT ERlangen (VAXSTER) at the chair for Crystallography and Structural Physics (Universität Erlangen-Nürnberg, Germany) using its former 30 W Cu Kα (λ = 1.5418 Å) Microfocus X-ray source GeniX with a FOX 2D parallel beam mirror optics (Xenocs, Sassenage, France). The system was equipped with a Cu Kα (λ = 1.5418 Å) Microfocus X-ray source (GeniX, 30 W, Xenocs, Sassenage, France). The beam was collimated by two automated double slit systems (aperture sizes 0.7 × 0.7 mm^2^ and 0.4 × 0.4 mm^2^) with a distance of about 1.2 m. The second slit system consists of four “scatter-less” silicon single crystal blades. The sample position was located within an evacuated detector tube. A 2D Pilatus 300 K detector (Dectris Ltd., Baden, Switzerland) was used to collect the scattered radiation. All measurements were performed at 22.2 °C. The collimation line was tilted and shifted with respect to the horizontal plane allowing grazing incidence angles which maximize the scattering volume and enhance the scattered intensity. The incidence angle α of 0.2° was chosen and a divergence of about 2.12% was obtained. α is smaller than the critical angle of total reflection of the substrate to limit the penetration depth and the scattering to the thin layer. The sample−detector distance (sdd) was calibrated to 78.3 mm using a silver behenate standard, providing a beam size of about 0.5 × 2 mm^2^ at the sample position. The width of the sample in the beam direction was only 2 mm to reduce peaks broadening due to the grazing incidence geometry. One-dimensional GIXD patterns were obtained by an angular average of the 2D patterns. The Ag thin film was exposed to iodine vapor with systematically varied times (0, 3, 6, 20, 40, 60, 180 and 1800 s) under standard conditions of temperature and pressure. Acquisition times were 3 hours for each of these exposure time points.

## Additional Information

**How to cite this article**: Bashouti, M. Y. *et al.* Systematic Surface Phase Transition of Ag Thin Films by Iodine Functionalization at Room Temperature: Evolution of Optoelectronic and Texture Properties. *Sci. Rep.* **6**, 21439; doi: 10.1038/srep21439 (2016).

## Supplementary Material

Supplementary Information

## References

[b1] Park H. K., Yoon J. K. & Kim K. Novel fabrication of Ag thin film on glass for efficient surface-enhanced Raman scattering. Langmuir 22, 1626–1629 (2006). 16460083 10.1021/la052559o

[b2] Xu Y. J., *et al.* Preparation of novel SiO_2_ protected Ag thin films with high reflectivity by magnetron sputtering for solar front reflectors. Sol.Energ. Mat. Sol. C 107, 316–321 (2012).

[b3] Kuroda, *et al.* Development of Ag alloy thin film with both high reflectance and adhesion for high density opt-electronic module. Ieee. T. Compon. Pack. T 30, 302–308 (2007).

[b4] Lee C. C., Chen S. H. & Jiang C. C. Optical monitoring of silver-based transparent heat mirrors. Appl. Optics 35, 5698–5703 (1996). 10.1364/AO.35.005698 21127578

[b5] Sahu D. R. & Huang J. L. High quality transparent conductive ZnO/Ag/ZnO multilayer films deposited at room temperature. Thin Solid Films 515, 876–879 (2006).

[b6] Bertran E., *et al.* RF sputtering deposition of Ag/ITO coatings at room temperature. Solid State Ionics 165, 139–148 (2003).

[b7] Wang Z. G., Chen Q. L. & Cai X. Metal-based transparent heat mirror for ultraviolet curing applications. Appl. Surf. Sci. 239, 262–267 (2005).

[b8] Kume T., Amano T., Hayashi S. & Yamamoto K. Attenuated Total-Reflection Spectroscopy of Ag-Sio2 Composite Films. Thin Solid Films 264, 115–119 (1995).

[b9] George R. & Harrington J. A. Infrared transmissive, hollow plastic waveguides with inner Ag-AgI coatings. Appl. Optics 44, 6449–6455 (2005). 10.1364/ao.44.006449 16252656

[b10] Ageev L. A. & Miloslavsky V. K. Photoinduced Effects in Light-Sensitive Films. Opt.Eng. 34, 960–972 (1995).

[b11] Patimisco P., *et al.* Low-Loss Hollow Waveguide Fibers for Mid-Infrared Quantum Cascade Laser Sensing Applications. Sensors-Basel 13, 1329–1340 (2013). 23337336 10.3390/s130101329 PMC3574738

[b12] Sui K. R., *et al.* Optical properties of AgI/Ag infrared hollow fiber in the visible wavelength region. Opt.Lett. 33, 318–320 (2008). 18278096 10.1364/ol.33.000318

[b13] Bledt C. M., Harrington J. A. & Kriesel J. M. Loss and modal properties of Ag/AgI hollow glass waveguides. Appl. Optics 51, 3114–3119 (2012). 10.1364/AO.51.003114 22695541

[b14] Matsuura K., Matsuura Y.& Harrington J. A. Evaluation of gold, silver, and dielectric-coated hollow glass waveguides. Opt. Eng. 35, 3418–3421 (1996).

[b15] Navarro-Cia M., *et al.* Modes in silver-iodide-lined hollow metallic waveguides mapped by terahertz near-field time-domain microscopy. J. Opt. Soc. Am. B 30, 127–135 (2013).

[b16] Zeng X., *et al.* Fabrication and characterization of AgI/Ag hollow fibers for near-infrared lasers. Opt. Laser Technol. 49, 209–212 (2013).

[b17] Bashouti M. Y., Tung R. T. & Haick H. Tuning the Electrical Properties of Si Nanowire Field-Effect Transistors by Molecular Engineering. Small 5, 2761–2769 (2009). 19771570 10.1002/smll.200901402

[b18] Bashouti Y. M., *et al.* Heterojunction based hybrid silicon nanowire solar cell: surface termination, photoelectron and photoemission spectroscopy study. Prog. Photovolt: Res. Appl. 22, 1050–1061 (2014).

[b19] Talebi R., A. Nahal, Bashouti Y. M. & Christiansen S. Optical nano-structuring in light-sensitive AgCl-Ag waveguide thin films: Wavelength effect. Opt. Exp. 22, 30669–30682 (2015). 10.1364/OE.22.030669 25607015

[b20] Strydom C. A., van Staden J. F. & Strydom. J. An X-ray photoelectron spectroscopy investigation of silver iodide-coated ion-selective electrodes. Electroanalysis 4, 969- 973 (1992).

[b21] Cava R. J., Reidinger F. & Wuensch B. J. Single-crystal neutron-diffraction study of AgI between 23° and 300 °C. Sol. State Comm. 24, 411–416 (1997).

[b22] Wassermann H. J. & Vermaak J. S. On the determination of a lattice contraction in very small silver particles. Surface Science 22, 164 (1970).

[b23] Mohan D. B. & Sunandana C. S. Iodization of rf sputter induced disordered Ag thin films reveals volume plasmon-exciton "transition". J. Appl. Phys. 100, 064314 (2006).

[b24] Kumar P. S. & Sunandana C. S. gamma-AgI films by iodization at ambient temperature. Thin Solid Films 323, 110–114 (1998).

[b25] Kumar P. S. & Sunandana C. S. Strain-Induced Confinement of Excitons in Quasi-free AgI Nanoparticles. Nano Lett. 2, 431–434 (2002).

[b26] Kondo S., Itoh T. & Saito T. Strongly enhanced optical absorption in quench-deposited amorphous AgI films. Phys Rev B. 57, 13235–13240 (1998).

[b27] Guo Y. G., Lee J. S. & Maier J. AgI nanoplates with mesoscopic superionic conductivity at room temperature. Adv. Mater. 17, 2815–2819 (2005).

[b28] Schäfer J., Ristein J. & Ley L. & Ibach H. High Sensitivity photoelectron yield spectroscopy with computer-calculated electron optics. Rev. Sci. Instrum. 64, 653–658 (1993).

[b29] Fowler R. H. The Electron Theory of Metals. Nature. 126, 611–618 (1930).

[b30] Dweydari A. W. & Mee C. H. B. Work function measurements on (100) and (110) surfaces of silver. Phys Status Solidi A. 27, 223 (1975).

[b31] David L. R. CRC Handbook of Chemistry and Physics, CRC Press, Boca Raton, FL, (2005).

[b32] Mönch W., Semiconductor Surfaces and Interfaces, Vol. 26. (Eds: Ertl G., Gomer R., Mills D.) Springer, Berlin (1993).

[b33] Smith P. V. A tight-binding approach to the electronic structure of the silver halides—II: The silver iodide polymorphs. J. Phys. Chem. Solids 37, 589 (1976).

[b34] Ristein J., Mammadov S. & Seyller T. Origin of Doping in Quasi-Free-Standing Graphene on Silicon Carbide. Phys. Rev. Lett. 108, 246104 (2012). 23004296 10.1103/PhysRevLett.108.246104

[b35] Morgan B. J. & Madden P. A. Effects of lattice polarity on interfacial space charges and defect disorder in ionically conducting AgI heterostructures. Phys. Rev. Lett. 107, 206102 (2011). 22181747 10.1103/PhysRevLett.107.206102

[b36] Bernardini F., Fiorentini V. & Vanderbilt D. Spontaneous polarization and piezoelectric constants of III-V nitrides. Phys. Rev. B. 56, 10024 (1997).

[b37] Leckey R., Riley J., Cai Y., Faul J. & Ley L. How Successfully Does Angle-resolved Photoemission Determine the Band Structure of Semiconductors? Aust. J. Phys. 46, 717–728 (1993).

