# Supplementary material for: Systematic Surface Phase Transition of Ag Thin Films by Iodine Functionalization at Room Temperature: Evolution of Optoelectronic and Texture Properties
Source: Sci Rep. 2016 Feb 22;6:21439. doi: 10.1038/srep21439 (PMC4761880; doi:10.1038/srep21439)
Supplement: Supplementary Information [file srep21439-s1.doc]

**Supporting Information**

**Systematic Surface Phase Transition of Ag Thin Films by Iodine Functionalization at Room Temperature: Evolution of Optoelectronic and Texture Properties**

Muhammad Y. Bashouti,^1,*^ Razieh Talebi,^1,2^ Thaer Kassar,^3^ Arashmid Nahal,^4^ Jürgen Ristein,^5^ Tobias Unruh,^3^ Silke H. Christiansen ^1,6,7^

^1^ Max-Planck Institute for the Science of Light, Günther-Scharowsky-Str. 1, D-91058, Erlangen, Germany, ^2^ Department of Physics, Faculty of Science, University of Isfahan, Hezar Jerib, 81746-73441, Isfahan, Iran, ^3^ Lehrstuhl für Kristallographie und Strukturphysik, Friedrich-Alexander-Universität Erlangen-Nürnberg, Staudtstraße 3, 91058 Erlangen, Germany, ^4^ Department of Physics, Photonic Materials Research Laboratory, University of Tehran, 14399-55961, Tehran, Iran, ^5^ Department of Physics, Chair of Laser Physics, Universität Erlangen-Nürnberg, Staudtstr. 1, D-91058 Erlangen, Germany, ^6^ Institute of Nanoarchitectures for energy conversion, Helmholtz-Center Berlin (HZB), Hahn-Meitner-Platz 1, D-14109 Berlin, Germany, ^7^ Physics Department, Freie Universität Berlin, Arnimallee 14, 14195 Berlin, Germany

*** Corresponding Author:**

[Muhammad.Bashouti@mpl.mpg.de](mailto:Muhammad.Bashouti@mpl.mpg.de)

**1) GIXD detector images**

The GIXD detector images collected as different iodine exposure.


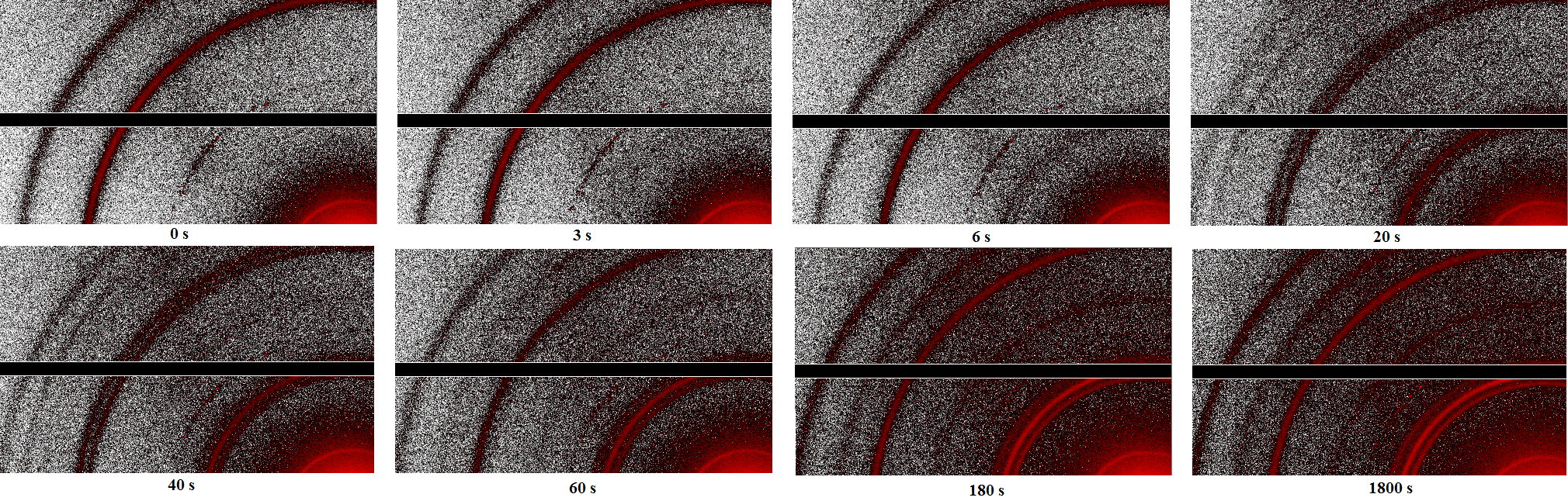


**Figure S1**. **GIXD patterns**. The GIXD patterns collected at systematically varied times of Ag thin film exposure to iodine vapor.

**2) Texture:**


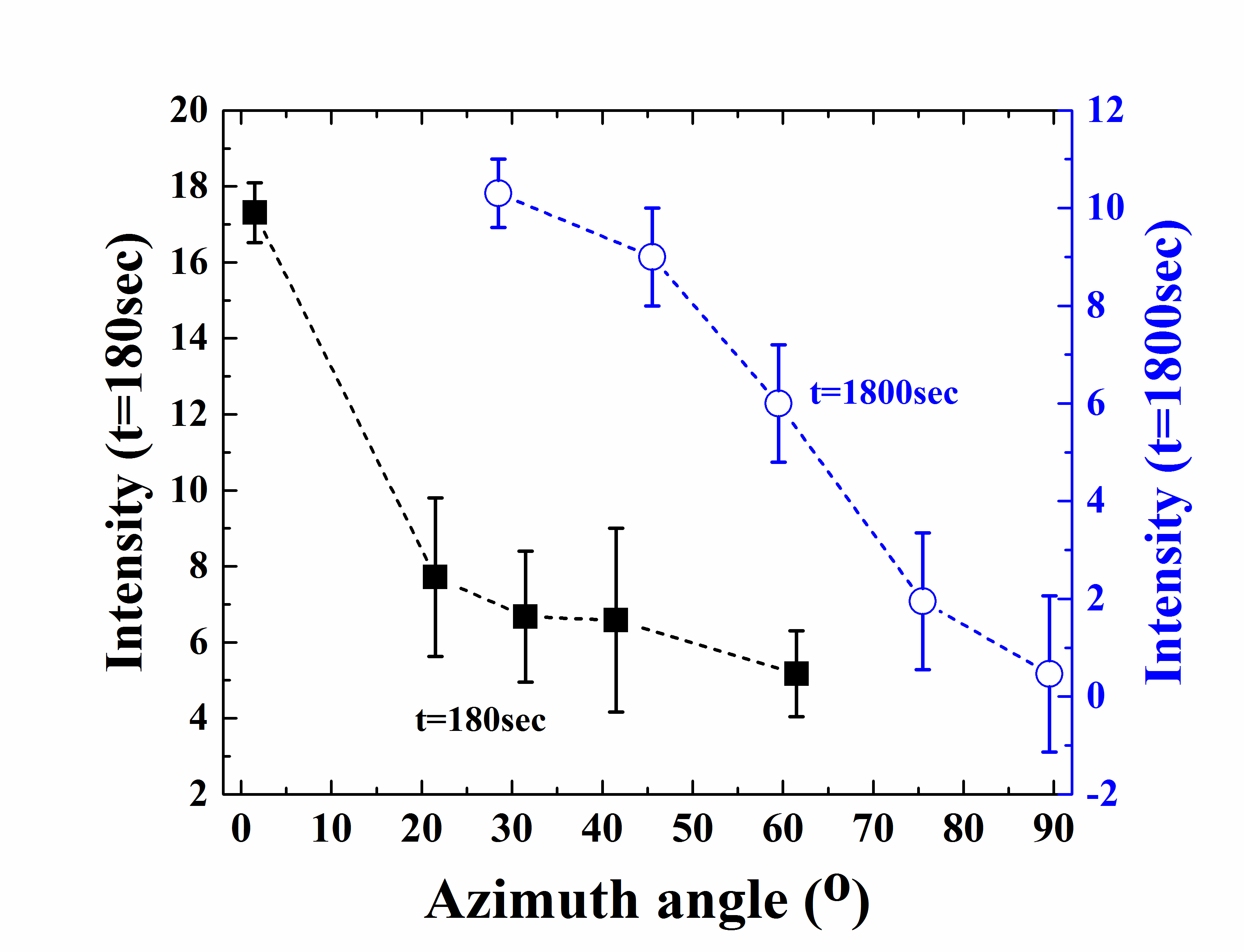


**Figure S2.** **Azimuthal distribution.** Azimuthal distribution of the intensity scattered from (100) planes of the β-AgI crystallites after an exposure to iodine for 180sec and 1800 s.

1. **XPS Survey:** Full survey of XPS on AgTF before (t=0) and after completed iodine exposure (t=180).


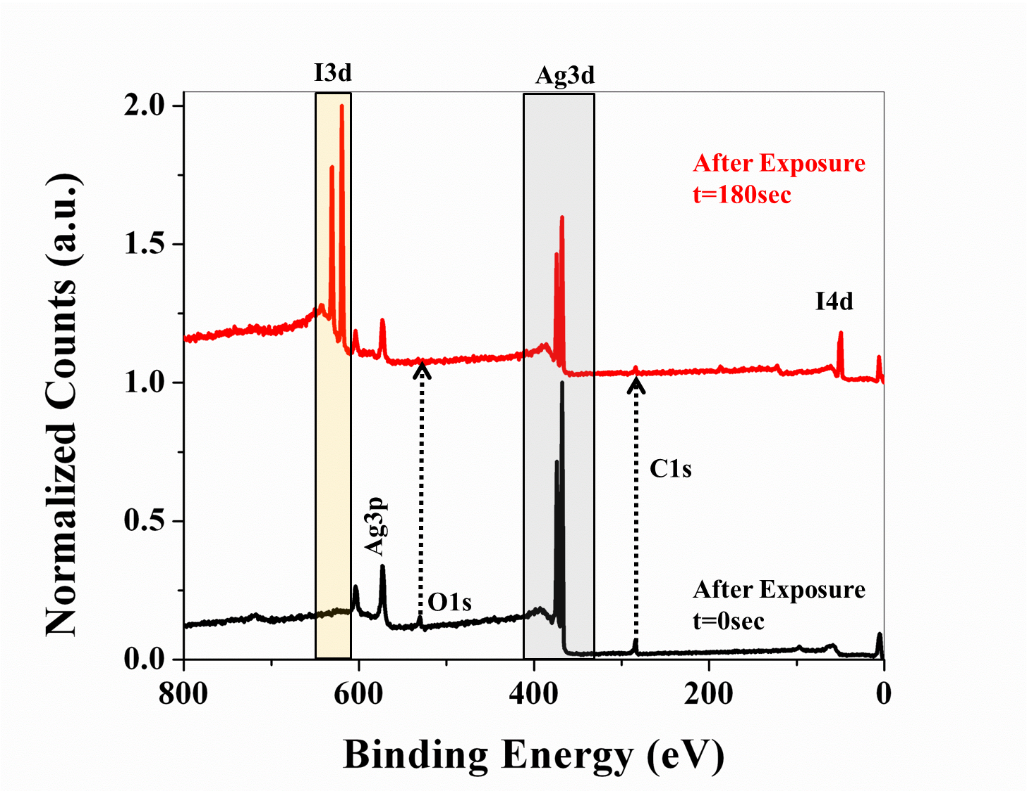


Figure S3. XPS spectra. XPS spectra of Ag TF before (t=0) and after Iodine exposure (t=180s).

**4) XPS deconvolution:** The intensity ratio of the formed Ag-I on the Ag TF was tracked by fitting two double orbit peaks of Ag_3d_ (i.e. Ag3d_3/2_ and Ag3d_5/2_) and I_3d_ (i.e. I3d_3/2_ and I3d_5/2_). The integrated area under the Ag_3d_ peak (sum of Ag_3d5/2_ and Ag_3d7/2_) was rationed to the one under the I_3d_ peak (sum of I_3d5/2_ and I_3d7/2_). The coverage of the Ag-I was normalized with the highest value obtained for Ag-I which is achieved after 180s. This term is expressed throughout the text as “R = (I_3d_ /Ag_3d_)_t_/(I_3d_ /Ag_3d_)_t=180s_” and shown in Figure 2a.


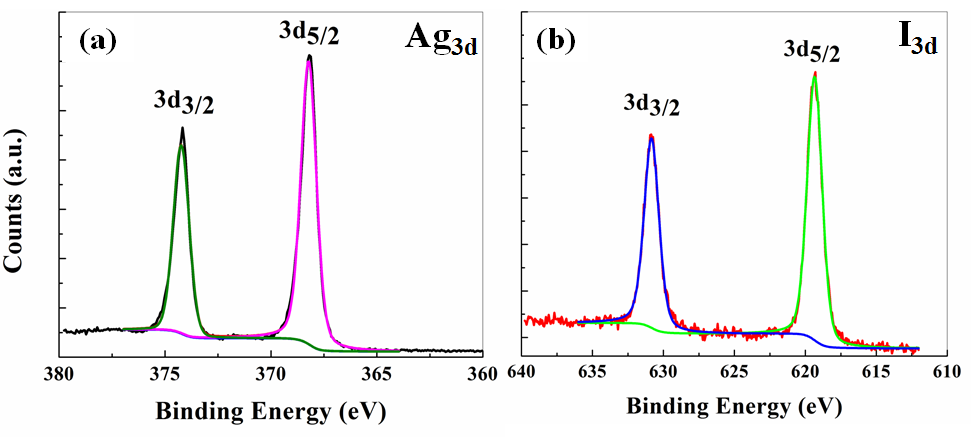


Figure S4. XPS deconvolution. XPS deconvolution with High resolution of (a) Ag3d and,(b) I3d.

**Table S1.** **Binding Energy of Ag 3d and I3d.** Summary of the deconvoluted XPS peaks of Ag_3d_ and I_3d_ along the Iodine exposure time. The error in the binding energy positions and FHWM is ±0.02eV.

| **Time (sec)** | **Ag_3d_** | | | **I_3d_** | | |
| --- | --- | --- | --- | --- | --- | --- |
|  | **Ag_3d_**_5/2_ (eV) | **Ag_3d_**_7/2_ (eV) | **FHWM** (eV) | **I_3d_**_5/2_ (eV) | **I_3d_**_7/2_ (eV) | **FHWM** (eV) |
|  |  |  |  |  |  |  |
| 0 | 368.14 | 374.14 | 0.815 | -- | -- | -- |
| 3 | 368.19 | 374.19 | 0.840 | 619.10 | 630.57 | 1.245 |
| 6 | 368.17 | 374.18 | 0.835 | 619.07 | 630.54 | 1.205 |
| 20 | 368.22 | 374.23 | 0.875 | 619.34 | 630.84 | 1.255 |
| 40 | 368.30 | 374.31 | 0.905 | 619.45 | 630.94 | 1.145 |
| 60 | 368.31 | 374.33 | 0.940 | 619.49 | 630.97 | 1.145 |
| 120 | 368.34 | 374.35 | 0.935 | 619.51 | 631.00 | 1.135 |
| 180 | 368.35 | 374.36 | 0.935 | 619.51 | 630.98 | 1.145 |
|  |  |  |  |  |  |  |

**5) AFM:**

**
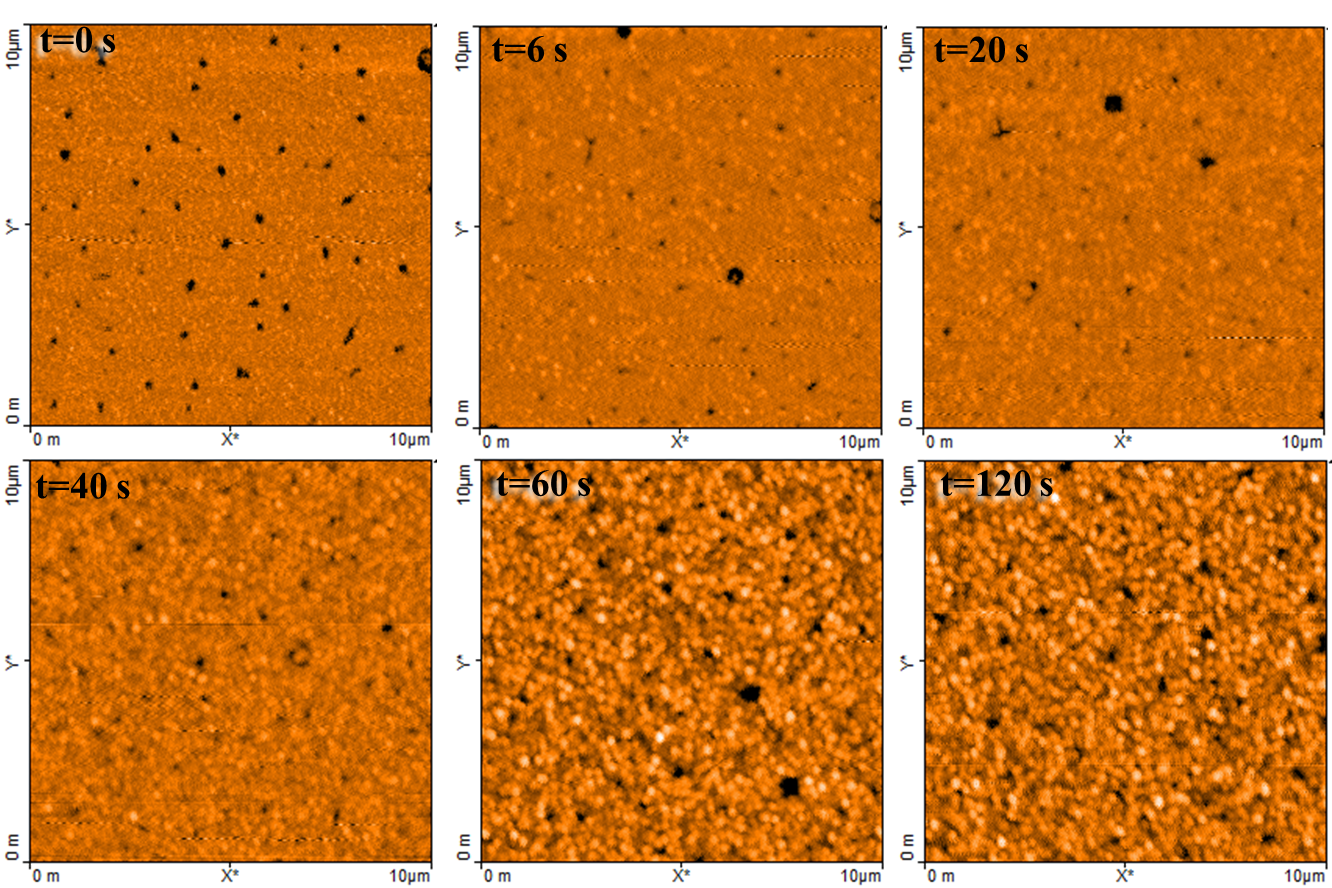
**

Figure S5. AFM images of the AgTF along iodine exposure. AFM images of the AgTF surface along the Iodine exposure. We did not include the AFM after 3s and 180s since they looks identical to 6s and 180s respectively.

**Table S2. Thickness of the AgTF along the Iodine exposure**. Thickness of the AgTF along the Iodine exposure as obtained from AFM.

| **Time (sec)** | **Thickness (nm)** | **Developed AgI (nm)** |
| --- | --- | --- |
| 6 | 11.39 | 1.39 |
| 20 | 14.62 | 3.23 |
| 40 | 21.23 | 6.61 |
| 60 | 31.19 | 9.96 |
| 120 | 45.21 | 14.02 |

For the initial iodine exposure time (0-60sec), the thickness of the AgI was calculated by the two layer model as shown in Figure 4a. After 60sec, the thickness of the AgI and Ag can be followed by AFM. The AFM images of Ag TF before and after iodization are indicated in supplementary information (Figure S5 and Table S2). The images show an increased thickness of the whole thin film (i.e Ag and AgI) as function of the iodine exposure. The increased thickness is related to the increased thickness of the developed AgI on the Ag thin film. Roughly speaking, the increased thickness of AgI after each iodine exposure can be calculated by subtraction the last layer thickness from the previous layer. For example, the thin film thickness increased from 31.19nm to 45.21nm after exposure 60s and 120s respectively, thus, the developed layer AgI is 14.02nm. However, we could not measure the real thickness of the Ag and AgI thin layers separately and we based on modeling XPS at the initial exposure time (0-60sec) and AFM (0-120sec) which supported by the optical density spectrum at 420nm. Simulations shows that the area under the absorption peak at 420nm shows the same trend of the developed AgI layer which found in AFM. [Berry, R. C. Structure and Optical Absorption of AgI Microcrystals. *Phys. Rev.* **161**, 848-851, (1967)].

**6) Optical density of the initial Ag TF before iodine exposure:**


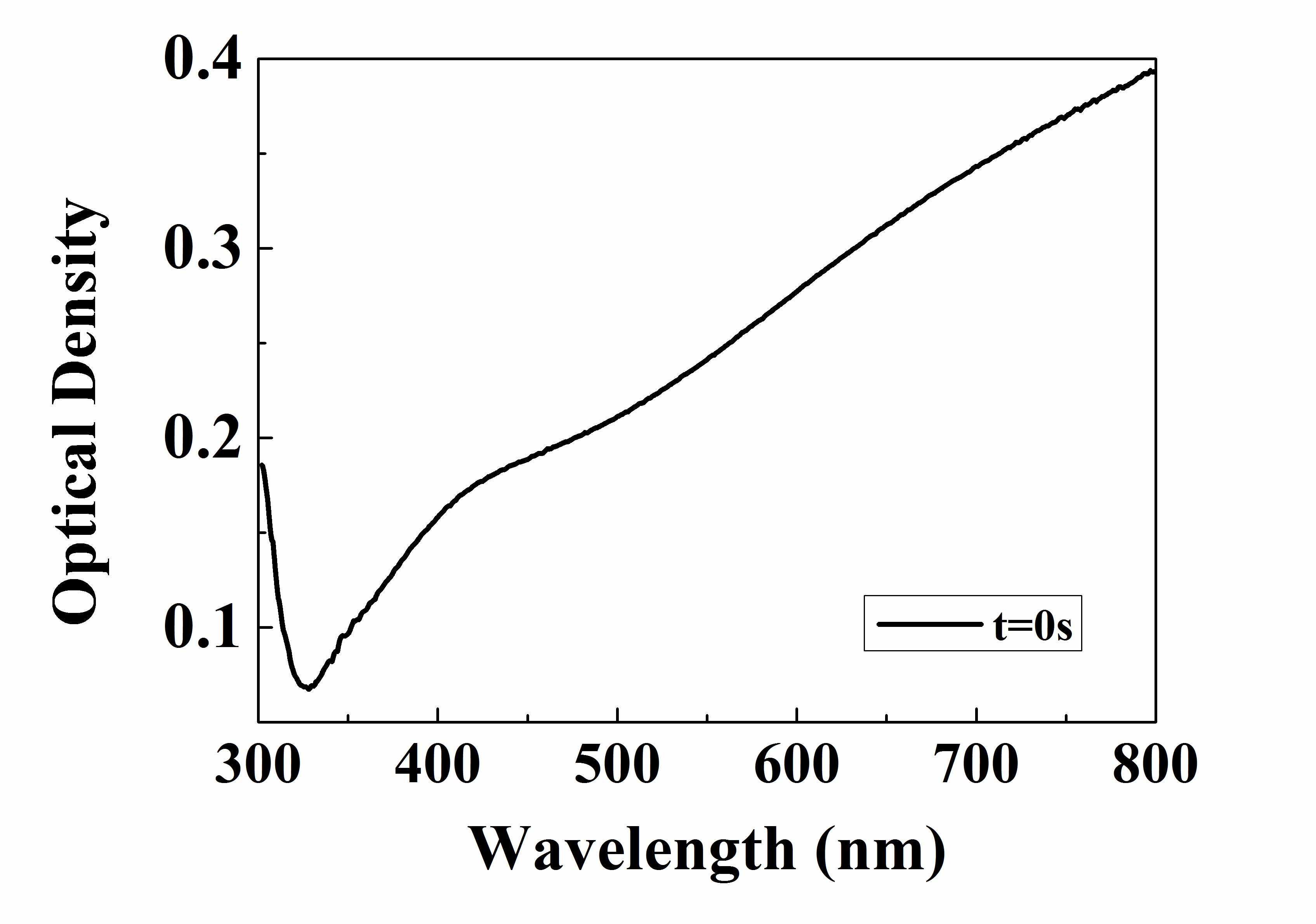


Figure S6. Optical density. Optical density spectrum of AgTF at t=0s.

**7)** **Picture of the AgTF and AgI**


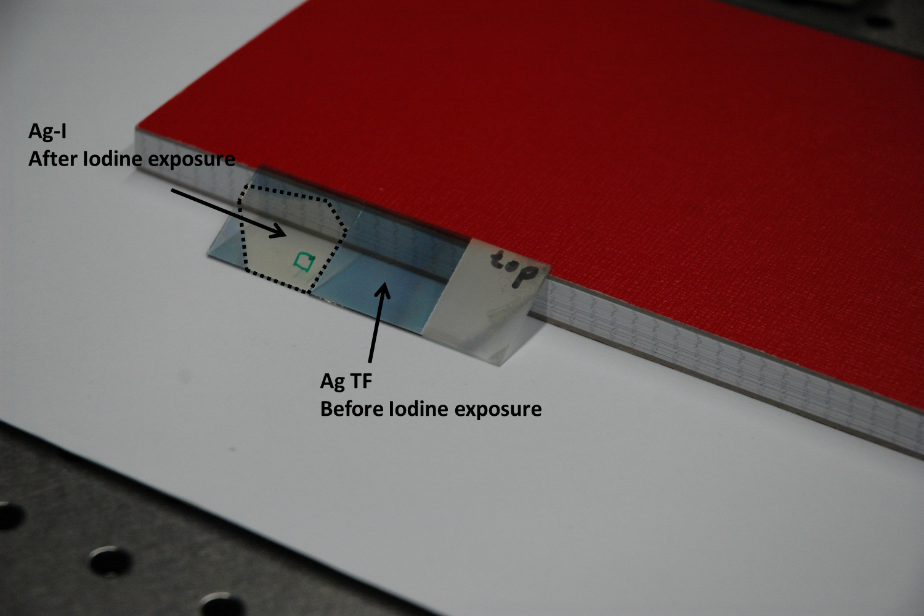


Figure S7. Optical image. Picture of Ag TF before and after Iodine exposure shows the transparency of both. As shown, the Ag TF becomes more transparent after exposing to iodine.

**8) Work Function:**

**Table S3. Work function. The absolute values of the work function of the AgTF along the Iodine exposure.**

| **Time (sec)** | **Absolute Work Function (eV)** |
| --- | --- |
| 0 | 4.631 |
| 3 | 5.015 |
| 6 | 5.055 |
| 20 | 5.182 |
| 40 | 5.321 |
| 60 | 5.392 |
| 120 | 5.466 |
